# Effects of immobilization mask material on surface dose

**DOI:** 10.1120/jacmp.v6i1.1957

**Published:** 2005-03-17

**Authors:** Scott W. Hadley, Robin Kelly, Kwok Lam

**Affiliations:** ^1^ Department of Radiation Oncology Physics The University of Michigan Box 0010, 1500 E. Medical Center Drive Ann Arbor Michigan 48109 U.S.A.

**Keywords:** surface dose, thermoplastic mask, skin sparing effect

## Abstract

This work investigates the increase in surface dose caused by thermoplastic masks used for patient positioning and immobilization. A thermoplastic mask is custom fit by stretching a heated mask over the patient at the time of treatment simulation. This mask is then used at treatment to increase the reproducibility of the patient position. The skin sparing effect of mega‐voltage X‐ray beams can be reduced when the patient's skin surface is under the mask material. The sheet of thermoplastic mask has holes to reduce this effect and is available from one manufacturer with two different sizes of holes, one larger than the other. This work investigates the increase in surface dose caused by the mask material and quantifies the difference between the two samples of masks available. The change in the dose buildup was measured using an Attix parallel plate chamber by measuring tissue maximum ratios (TMRs) using solid water. Measurements were made with and without the mask material on the surface of the solid water for 6‐MV and 15‐MV X‐ray beams. The effective thickness of equivalent water was estimated from the TMR curves, and the increase in surface dose was estimated. The buildup effect was measured to be equivalent to 2.2 mm to 0.6 mm for masks that have been stretched by different amounts. The surface dose was estimated to change from 16% and 12% for 6 MV and 15 MV, respectively, to 27% to 61% for 6 MV and 18% to 40% for 15 MV with the mask samples.

PACS number: 87.53.Dq

## I. INTRODUCTION

Treatment of cancers in the head and neck require a high level of accuracy in the positioning of the patient for daily treatments. Special thermoplastic head and neck immobilization devices are used to reproducibly position the patient on the table. It is possible for the thermoplastic mask to increase the surface dose to the patient and compromise the skin sparing effect of high‐energy X‐ray beams.

Increase in surface dose caused by devices and materials used during treatment delivery has been measured by several authors.^(1)^ ^,^ ^(2)^ Recently Lee et al. measured an average 18% increase in surface dose caused by thermoplastic masks used for intensity‐modulated radiotherapy treatments.^(3)^ Higgins et al. measured the increase in surface dose caused by carbon fiber tables to be as much as 400%.^(4)^ Thermal plastic mask material can increase the effective depth of the patient's skin and thus increase the dose the skin receives.

Our clinic noticed an earlier onset of skin toxicity of head and neck patients since implementing the Sinmed patient positioning and immobilization system. This system uses a mask material that is heated to about 80 °C and then stretched over the patient to make a form‐fitting custom mask. This mask snaps into a carbon fiber base to increase the reproducibility of the positioning. Sinmed makes available two different versions of thermoplastic mask, one with small holes and the other with larger holes.

In this work we measure the change in dose buildup caused by the thermoplastic mask and quantify the difference between mask materials available from Sinmed. The buildup region was characterized by measuring tissue maximum ratios (TMRs) from a depth of 0 to the depth of maximum dose of the beam. When the mask is made, different parts of it are stretched by different amounts. Several different samples were measured that were stretched by different amounts to obtain a range of possible results. The manufacturer reports that the unstretched material represents 1.1 mm equivalent depth and causes 55% and 46% surface dose relative to maximum dose of the beam for 6‐MV and 8‐MV beams, respectively.

## II. MATERIALS AND METHODS

### A. Mask material samples

The mask material changes its size and thickness and thus its effect on the buildup region when it is stretched over the patient. Samples of the mask were stretched by different amounts, and the buildup effect was measured to quantify these changes. The mask was stretched in a reproducible way by heating a 10×10cm section of mask material and stretching it over forms of size 15×15cm, 20×20cm, and 25×25cm. Once they had cooled, 11×11cm sections were cut out of the center and used for testing. These samples were tested along with unstretched samples of mask for the material with both large and small holes, resulting in eight different samples to be tested using 6‐MV and 15‐MV X‐rays of a Varian 21Ex LINAC.

**Figure 1:**
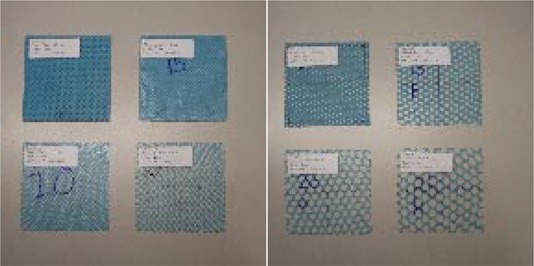
Photographs of the mask samples measured in this investigation. The photo on the left is the material with the smaller holes, and the photo on the right is the material with the larger holes. The material was stretched different amounts in a reproducible way.

The physical changes in the mask were measured as well. The actual change in the area of the samples was estimated by drawing a 10×10cm square on the sample before stretching and then measuring it after it had been stretched. The size of the form the sample was stretched over is reported as the percent area increase‐nominal and the measured increase as the percent area increase‐measured. The area density of the mask was determined by dividing the mass of the mask sample by its area (121 cm^2^) and reported as grams per square centimeter. The thickness was measured using a caliper. Figure 1 shows the mask samples used in this investigation.

### B. TMR measurements

The buildup effect of the mask material was determined by measuring TMRs with and without the mask on the surface of a solid water phantom. Tissue maximum ratios were measured using an Attix parallel plate chamber placed 100 cm from the X‐ray source of a 10×10cm field. Measurements were made at depths of 0 mm, 2 mm, 3 mm, 4 mm, 5 mm, 8 mm, 10 mm, 15 mm, and 30 mm of solid water with and without the mask material placed on top. The reproducibility of the Attix chamber allowed for a single measurement to be made at each depth with periodic consistency checks to ensure that the chamber response did not change during measurements. The measurements with the 6‐MV beam were normalized at its $d_{\max}$ of 15 mm and the 15‐MV beam at its $d_{\max}$ of 30 mm. All depths reported in the TMRs represent the depth of solid water excluding the thickness of the material when the mask was present.

### C. Equivalent thickness of water

The presence of the mask material will shift the position of the TMR curve horizontally to the left by an amount equal to the equivalent thickness, $d_{e}$, of solid water that the mask represents. ${\text{TMR}}_{0}$ represents the TMR obtained with no mask material in the beam, and ${\text{TMR}}_{m}$ the TMR with the mask present but normalized using the measurement at $d_{\max}$ with no mask material present. The horizontal distance between points of equal TMR,

$${\text{TMR}}_{m}(d_{m})={\text{TMR}}_{0}(d_{0}),$$

was used to estimate $d_{e}$. The depths $d_{m}$ and $d_{0}$ for several different points before $d_{\max}$ were used in a linear correlation to determine $d_{e}$. The *y*‐intercept of the best‐fit line through the points $d_{m}$ and $d_{0}$ is taken to be $d_{e}$; see Fig. 4. The steepest part of the TMR curve was used for the linear regression. This meant that for 6‐MV X‐ray measurements, the data from depths of 0 mm to 8 mm were used, and for 15‐MV X‐rays, depths of 0 mm to 10 mm were used.

**Figure 2:**
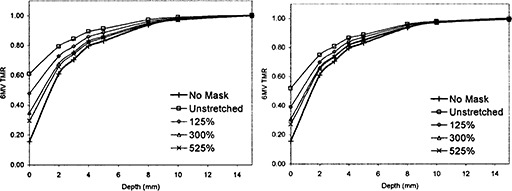
Buildup effect of mask material for 6‐MV X‐rays. The graph on the left is the buildup effect for the mask with smaller holes, and the graph on the right is for the mask with larger holes. The uppermost curve is the unstretched mask, and the curve on the bottom is with no mask present. As the mask was stretched more, the TMR curve moved from the unstretched curve to the curve with no mask.

**Figure 3:**
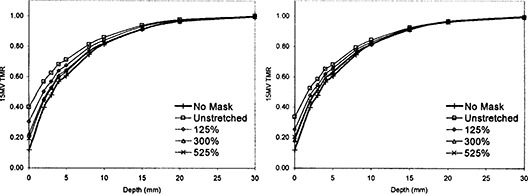
Buildup effect of mask with 15‐MV X‐rays. The graph on the left is for the mask with smaller holes and for larger holes on the right. The uppermost curve is the unstretched mask, and the curve on the bottom is for no mask material. As the mask is stretched, its buildup effect is reduced.

**Figure 4:**
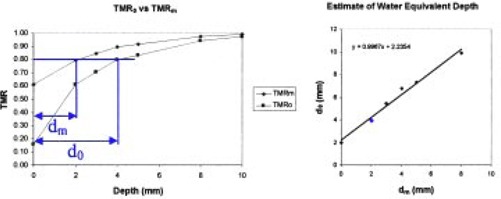
Example of the linear regression used to estimate the thickness of water the buildup of the mask represents. The depths of equal TMR on the two curves are used to determine the equivalent depth of water the mask represents. The second point on ${\text{TMR}}_{0}$ is used as an example and is highlighted in blue.

### D. Surface dose measurements

The increase in the surface dose caused by the presence of mask was compared by looking at the TMR at depth 0 with and without the mask samples. The Attix chamber is designed to have minimal overresponse due to in‐scatter from the side wall material of the chamber and a measurement depth of 0 from the outer surface.^(5)^ ^,^ ^(6)^ The surface dose is reported as a ratio of the maximum dose to the dose at depth 0.

The size of the holes in some of stretched samples was comparable to the Attix chamber collector diameter of 1.27 cm. It was conceivable that different amounts of mask material overlap the collector if the sample were shifted. To test for this, the sample with the largest holes after being stretched was measured at depth 0 in six different positions: four linear shifts, one with a hole centered on the chamber, and one with the web centered on the chamber. These measurements were compared to the average reading and reported as a percent difference from the average.

## III. RESULTS

### A. Physical changes

The physical changes in the mask material are summarized in Table 1. This table summarizes the changes in the size of the mask as well as the decreased area density as a result of stretching. There was little difference between the way the mask with small or large holes stretched. Each sample stretched to about 80% of the nominal value. The area density of the mask material decreased as the stretching increased. The mask with larger holes was 24% less dense than the mask with the smaller holes for the unstretched samples.

**Table 1:** Physical changes in mask material due to stretching. The masks were stretched over forms designed to increase the area by a known amount.

	% Area increase—nominal	% Area increase—measured	Area density $\left(g/{\text{cm}}^{2}\right)$	Thickness (mm)
	0%	0%	0.225	2.39
small holes	125%	82%	0.131	1.75
mask material	300%	224%	0.076	1.24
	525%	417%	0.053	1.17
	0%	0%	0.170	2.16
large holes	125%	103%	0.096	1.78
mask material	300%	252%	0.058	1.42
	525%	441%	0.047	1.24

### B. Change in dose buildup

Figures 2 and 3 show the TMRs for the mask material for 6‐MV and 15‐MV X‐rays, respectively. The uppermost TMR curve is for the mask with no stretching, and the lowermost is the ${\text{TMR}}_{0}$ for no mask at all. The TMR curves show that the more the mask material was stretched, the smaller the buildup effect. The mask with the larger holes showed a reduced buildup effect compared to the mask with smaller holes.

### C. Equivalent thickness of water

Figure 4 shows an example of the liner regression used to quantify the shift in the TMR curves and estimate the effective water equivalent thickness, $d_{e}$, of the mask. Table 2 contains the measured thickness $d_{e}$ for the various samples of mask for 6‐MV and 15‐MV X‐rays. The results for 6‐MV and 15‐MV X‐rays agree well with one another. The mask samples represent between 2.4 mm and 1.2 mm of solid water. The mask with the larger holes has a decrease in $d_{e}$ anywhere between 25% to 15% over the mask with the smaller holes.

**Table 2:** Estimates of the effective thickness of water each mask represents for 6‐MV and 15‐MV X‐rays. The area density and thickness of the mask are presented for comparison.

	% Area increase‐nominal	$d_{e}$ (mm) 6 MV	$d_{e}$ (mm) 15 MV	Area density $\left(g/{\text{cm}}^{2}\right)$	Thickness (mm)
small	0%	2.24	2.21	0.225	2.39
holes	125%	1.36	1.32	0.131	1.75
mask	300%	0.77	0.80	0.076	1.24
	525%	0.59	0.60	0.053	1.17
large	0%	1.67	1.67	0.170	2.16
holes	125%	0.97	1.02	0.096	1.78
mask	300%	0.62	0.65	0.058	1.42
	525%	0.46	0.51	0.047	1.24

### D. Surface dose increase

The increase in the surface dose caused by the mask was compared to the TMR measurements at depth 0 without the mask samples over the chamber. Table 3 summarizes the increase in surface dose caused by the mask samples. The mask samples with smaller holes had TMR increase to 0.27–0.61 from 0.16 with no mask. The mask with the larger holes had surface TMR increase to 0.18–0.40 from 0.12 at depth 0.

**Table 3:** Estimates of the surface dose relative to $d_{\text{max}}$ each mask causes for 6‐MV and 15‐MV X‐rays. The area density and thickness of the mask are presented for comparison.

	% Area increase‐ nominal	Surface dose 6 MV	Surface dose 15 MV	Area density $\left(g/{\text{cm}}^{2}\right)$	Thickness (mm)
no mask		16%	12%		
small	0%	61%	40%	0.225	2.39
holes	125%	48%	30%	0.131	1.75
mask	300%	35%	22%	0.076	1.24
	525%	29%	19%	0.053	1.17
large	0%	52%	34%	0.170	2.16
holes	125%	39%	25%	0.096	1.78
mask	300%	31%	20%	0.058	1.42
	525%	27%	18%	0.047	1.24

The sample with the large holes that was stretched the most was shifted to several different positions to test for any variation in the dose as different amounts of mask overlapped the detector. These tests showed the variation from the average TMR at depth 0 for this sample to be ±1%.

## IV. DISCUSSION

The effective thickness of water represented by the buildup effect of the mask was found to be dependent on how much the mask was stretched and whether the mask were made using the material with small or large holes. The increased dose in the buildup region is caused by the additional mass of plastic over the Attix chamber. As the samples were stretched, the change in the measured dose could have resulted from the increased size of the holes. The measured dose was a combination of the area under the holes and the area under the mask. The measurements presented here showed that the best way to reduce this mass over the patient's skin is to use the mask material with the larger holes.

Stretching the mask more reduces the buildup effect of the mask and decreases the surface dose. When making the mask there is little control over how much the material is stretched. Overstretching the mask to reduce the buildup effect could compromise the rigidity of the mask and reduce its ability to aid in patient positioning.

The density of the mask material compares well with that of water. When the area density is divided by the equivalent thickness of water $d_{e}$, the mass densities range from $0.88\;\text{to}1.02\;g/{\text{cm}}^{3}$. The manufacturer‐stated water equivalent of 1.1 mm for an unstretched sample of the mask is less than that measured in this study. The surface dose of 55% for a 6‐MV beam stated by the manufacturer is consistent with this study. Comparisons between these results will depend on the LINAC used in the study.

## V. CONCLUSION

The purpose of this work was to measure the change in the dose buildup and surface dose increase of a common thermoplastic mask material used for head and neck immobilization. Our measurements were made on the Sinmed thermal plastic mask. This mask comes in two versions: one with small holes and another with larger holes. The buildup effect of the thermoplastic mask was reduced as the mask was stretched more. The plastic mask material was found to be roughly water equivalent when the equivalent thickness of water is compared to the area density.

Using a mask with larger holes will reduce the surface dose compared to the mask with the smaller holes. As the material is stretched more, the advantage is decreased. Under the best conditions the mask increased the surface dose by a factor of 1.5 and at worst a factor of 3.8. Our clinical experience is that the masks made for patients are stretched an amount consistent with a nominal increase in area of 125%. Different parts of the mask will stretch much more than others due to widely varying body contours around the neck and shoulders. At our institution the clinic has taken up the practice of cutting out the sections of mask where beams enter most tangentially to avoid the dose increase.

## ACKNOWLEDGMENT

This work was sponsored by NIH P01‐CA59827.
